# Color of Colon Content of Normal and Intrauterine Growth-Restricted Weaned Piglets Is Associated with Specific Microbial Taxa and Physiological Parameters

**DOI:** 10.3390/ani10061073

**Published:** 2020-06-22

**Authors:** Maria Wiese, Yan Hui, Dennis S. Nielsen, Andrew R. Williams, Julie C. Lynegaard, Nicolai R. Weber, Charlotte Amdi

**Affiliations:** 1 Department of Food Science, Faculty of Science, University of Copenhagen, DK-1958 Frederiksberg C, Denmark; huiyan@food.ku.dk (Y.H.); dn@food.ku.dk (D.S.N.); 2 Department of Veterinary and Animal Sciences, Faculty of Health and Medical Sciences, University of Copenhagen, DK-1870 Frederiksberg C, Denmark; arw@sund.ku.dk (A.R.W.); julie.lynegaard@sund.ku.dk (J.C.L.); nirw@seges.dk (N.R.W.); ca@sund.ku.dk (C.A.); 3 Present address: Pig Research Centre, Danish Agriculture and Food Council, Axeltorv 3, DK-1609 Copenhagen V, Denmark

**Keywords:** gut microbiota, intrauterine growth-restricted, piglets, fecal color

## Abstract

**Simple Summary:**

Selection for hyperprolific sows has increased litter sizes but has also increased the number of small piglets per litter. A large percentage of these piglets have been exposed to intrauterine growth restriction (IUGR) during gestation, and this is accompanied by higher mortality and reduced growth in pig production. In humans, IUGR is associated with long-term health consequences such as cardiovascular disease, as well as metabolic diseases such as type 2 diabetes. It is therefore of interest to study the gut microbiota (GM) of IUGR compared to normal piglets, as a well-balanced GM is associated with improved health outcomes. Differences in feces color was associated with different metabolite signatures and specific GM signatures. Understanding these differences in the composition of the microbial community and its functional capacity during weaning is important for pig production, as the GM play important roles in pig health and growth performance.

**Abstract:**

A well-balanced gut microbiome is associated with improved health outcomes, but to date, the GM of IUGR piglets have only been sparsely investigated. Here, we investigated GM composition, color of colon content, and blood parameters of 20 IUGR and 20 normal 24-day-old piglets. No significant differences were detected in colon microbiota composition between IUGR and the normal piglets with respect to alpha and beta diversity measures. The colon content of these piglets displayed three colors: brown, black, and yellow. Interestingly, the color of the colon content varied with microbial community composition, with significant differences in the relative abundance of taxa belonging to Fusobacteria and Treponema. Fusobacteria were most abundant in yellow fecal samples, with a mean relative abundance around 5.6%, whereas this was 0.51% within brown and 0.02% for the black fecal samples. Fusobacteria positively correlated with total blood protein, albumin, and triglycerides. Contrarily, *Treponema* was at 0.9% the most abundant in black fecal samples, while present at 0.1% of relative abundance in brown fecal samples and 0.01% in yellow samples, correlating positively with blood iron content. This study indicates that colon/fecal content color can be used as indicator for specific GM and metabolite signatures.

## 1. Introduction

Intrauterine growth restriction (IUGR) is defined as the impaired development of the mammalian fetus or its organs during gestation. The main cause of IUGR is an insufficiency of the placenta in distributing enough nutrients and oxygen to the offspring [1]. IUGR is a significant problem in human neonatology, affecting approximately 5–10% of human neonates, where it also predisposes for later development of, for instance, cardiovascular disease [2], as well as postnatal diabetes and impaired insulin action [3].

It is also a large problem in pig production, affecting approximately 15–20% of piglets [4,5,6]. Breeding selection for increased litter size has resulted in increased number of small piglets at birth, in particular those affected by IUGR [7]. Moreover, this condition may increase the risk of neonatal morbidity and affect the piglets’ postnatal growth performance [8,9]. Studies have also reported the predisposition to necrotizing enterocolitis in preterm growth-restricted neonates [10,11], even though conflicting results have been reported [12]. The intestinal microbiota of IUGR piglets could play a role in specific predispositions; its initial composition is likely to be altered by IUGR, as it delays perinatal intestinal development and strongly interacts with intestinal physiology [13]. Only a few studies have investigated the gut microbial community structure of IUGR mammals. Fança-Berthon et al. [13] found that IUGR not only modifies the cecocolonic microbiota in neonatal rats, but also affects short-chain fatty acid production in young adult rats. In adulthood, rats with IUGR still differed from controls, harboring fewer *Bifidobacterium* spp. at day 40 and more bacteria related to *Roseburia intestinalis* at day 100 [13].

More recently, Zhang et al. [14] identified alterations of small intestinal microbiota in IUGR piglets at 7, 21, and 28 days of age. Huang et al. [15] have reported on the characteristics of the gut microbiota (GM) colonization, inflammatory profile, and plasma metabolome in IUGR piglets during the first 12 h after birth. An increased abundance of *Escherichia*/*Shigella* and decreased abundance of *Clostridium* in IUGR piglets was closely associated with the alterations of weight, intestinal morphology, inflammatory cytokines, and plasma metabolites for the first 12 h after birth. Huang et al. [15] concluded that an unbalanced GM mediated by IUGR contributes to the development of inflammation and metabolic diseases. Previously, stool consistency has been linked with specific gut microbial profiles [16], but whether other, visual traits of fecal matter, such as color is linked to specific gut microbial signatures and host phenotype remains poorly understood. However, stool color can aid in the evaluation of the health state of infants, for example by screening for biliary atresia [17,18,19]. Stool color is hence a potential indicator of the health status at early age, e.g., reflecting bile flow and similar physiological factors. Further insights and indicative power might be gained from investigating stool color also in relation to the microbial composition at early life, whereas at an older age, a complex diet might be masking color to health correlations.

In this study, our aim was to investigate GM composition and its correlation with physiological biomarkers and stool color in newly weaned IUGR and normal piglets (day 24). Therefore, we quantified the gut microbial communities of the large intestine as well as colon content color and several immunological and metabolic parameters in blood. 

## 2. Materials and Methods 

### 2.1. Ethical Approval 

The experiment was carried out with respect to ethical procedures for animal experimentation and with approval from the Danish Animal Experimentation Inspectorate, license number (2016-15-0201-01018).

### 2.2. Experimental Design

The pigs were from a commercial piggery with 1600 sows (Danish Landrace and Danish Yorkshire (Danbred sows)) and were a subsample of a larger growth trial involving 284 piglets from 64 different sows over a time period of 5 weeks (see Lynegaard et al. [9]). Piglets were visually graded and categorized as either IUGR or normal on the basis of their headshape at birth (modified after the characteristics from [20,21]). An IUGR piglet included one or more of the following characteristics: (a) steep, dolphin-like forehead; (b) bulging eyes; and (c) wrinkles perpendicular to the mouth. If none of the criteria were applied, the piglet was defined as normal. For each IUGR piglet identified in a litter, one corresponding normal piglet from the same litter was chosen and ear tagged, and gender was balanced for when possible (i.e., a male IUGR piglet and a male normal piglet or the same for female piglets were selected when present in the litter). Each week (for 4 consecutive weeks), 10 piglets were pre-weaned and brought into the experimental facilities for sampling at 24 days of age (5 IUGR and 5 normal, each the same age per week) for the purpose of this study. Of the normal piglets, 9 males and 11 females were selected, and of the IUGR piglets, 11 males and 9 females were selected.

The diets of sows and piglets are presented in [9]. Briefly, sows had ad libitum access to drinking water and were fed a commercially formulated wheat–barley–soybean meal mast diet (Vilomix A/S, Mørke, Denmark) according to Danish recommendations [22]. All farrowing pens were installed with automatic milk cups, where the piglets had access to milk replacer (Schils, Sittard, the Netherlands) from day 1. Three to four times daily from day 14, the piglets received a handful (approximately 75 g, ±20 g) of dry feed (Danish New Wean feed; Danish Agro, Karise, Denmark). 

### 2.3. Inspection of Colon Content Color

The colon content color was recorded by visual inspection, as was also done by Bekkali et al. [23], who used visual inspection and photography for the presentation of fecal colors found in their study “Infant Stool Form Scale: Development and Results”. Three colors were identified: yellow, brown, and black. Photographs were taken with a digital camera.

### 2.4. Blood and Tissue Sampling

On day 24, piglets were transported to research facilities at the University of Copenhagen and were blood sampled within 1 h after arriving to the facilities. Five blood samples were taken from each piglet (results presented in Amdi et al. [24]). Briefly, the first sample was taken in a 1.8 mL citrate stabilized tube for thromboelastografy (TEG) and fibrinogen analysis. The second and third samples were taken into heparinized tubes for the lipopolysaccharides (LPS) challenge on blood, and phenotyping of peripheral leukocytes by flow cytometry. The fourth sample was taken in a 4.0 mL tube (Ethylenediaminetetraacetic acid (EDTA)) for hematology (CBC/Diff/Retic), and finally the fifth sample was taken in a 4.0 mL tube for serum and spun down, with the serum frozen for later analysis of Insulin-like growth factor 1 (IGF-1) and biochemistry. The tubes for TEG were taken to a nearby lab for immediate processing, which was done likewise for the tubes for the LPS challenge. The hematology profile was analyzed on an Advia 2120 Hematology System (Siemens Healthcare Diagnostics, Tarrytown, NY, USA), and the serum analyses for biochemistry were assayed using an 108 Advia 1800 Chemistry System (Siemens Healthcare Diagnostics, Tarrytown, NY, USA). 

### 2.5. Flow Cytomtery and Cytokine Analysis 

Peripheral blood mononuclear cells (PBMC) were obtained from heparinized blood samples by differential centrifugation using histopaque 1.077 (Sigma-Aldrich). Flow cytometric analysis of T cell and B cell populations and production of Interleukin (IL)-8, IL-6, IL-1β, IL-10, and Tumor necrosis factor alpha (TNFα) from PBMC following LPS stimulation was assessed as described by Amdi et al. [24].

### 2.6. Sampling of Colon Content 

The piglets (*n* = 20 per type) were anaesthetized with an intramuscular injection of a Zoletil mix (Zoletil 50; Virbac, Kolding, Denmark) containing xylacin (Narcoxyl 20 mg/mL; MSD Animal Health, Ballerup, Denmark), ketamine (Ketaminol 100 mg/mL; MSD Animal Health), and butorphanol (Torbugesic 10 mg/mL; ScanVet, Fredensborg, Denmark), and were left covered in a pen filled with straw to achieve deep anesthesia. Pigs were then weighed on a digital scale, and dual-energy X-ray absorptiometry (DXA) scanned (Lunar Prodigy Advance; GE Healthcare, Chicago, IL, USA), providing readings of body fat and muscle mass (presented in Lynegaard et al. [9]). The piglets were euthanized with an intracardial injection of 2 to 3 mL pentobarbital (200 mg/mL), and organs were removed and the total colon content was removed. The color of the colon contents differed and was hence recorded on the basis of three categories—black, brown, and yellow. 

### 2.7. Extraction of Total Colon Content DNA 

Colon content was homogenized with 1 M phosphate buffered saline (PBS), in a stomacher bag for 2 × 60 s using the Stomacher (Stomacher 400; Seward, Worthing, United Kingdom) at normal speed. Genomic DNA was extracted from the pellet of 1 mL fecal slurry using the Power Soil Kit protocol (MoBio Laboratories, Carlsbad, CA, USA). The FastPrep bead-beating step was performed in 3 cycles of 15 s each at a speed of 6.5 M/s in a FastPrep-24 Homogenizer (MP). DNA quantity and quality were measured using a NanoDrop 1000 (Thermo Scientific, Waltham, MA, USA). 

### 2.8. 16S rRNA Gene Library Preparation 

The colon microbiota composition was determined using tag-encoded 16S rRNA gene NextSeq-based (Illumina, CA, USA) high throughput sequencing. The V3 region of the 16S rRNA gene was amplified using primers compatible with the Nextera Index Kit (Illumina, San Diego, CA, USA) NXt_338_F: 5′-TCGTCGGCAGCGTCAGATGTGTATAAGAGACAGACWCCTACGGGWGGCAGCAG-‘3 and NXt_518_R: 5′-GTCTCGTGGGCTCGGAGATGTGTATAAGAGACAGATTACCGCGGCTGCTGG-‘3 [25] the PCR reactions and library preparation was conducted as described in Krych et al. [26]. 

### 2.9. High Throughput Sequencing and Data Integration 

For bioinformatic processing, the raw sequencing reads were merged and trimmed. Chimeras were removed and zero-radius operational taxonomic units (zOTUs) were constructed using UNOISE algorithm implemented in Vsearch [27,28]. Greengenes (version 13.8) database was used as reference for classification. Qiime2 [29] (2019.04) was used to process the forward analysis. Rare zOTUs with frequency below 0.1% of the mean library size were removed and filtered. zOTU table was rarified to adequate sample depth (65,000 counts) for alpha (Shannon index) and beta diversity calculation. In order to ensure consistent sequencing depth, we removed samples without adequate library size (<100 counts), which resulted in *n* = 19 and 17, respectively, for IUGR and NORMAL groups. 

### 2.10. Statistical Analysis

For pairwise comparison of alpha diversity index, we applied the Wilcoxon rank test. Principal coordinate analysis (PCoA) was conducted on unweighted and weighted Unifrac distance, permanova was performed to detect statistical difference between groups, and *p*-values were adjusted after pairwise tests. For zOTU enrichment analysis, we adopted Deseq2 to identify enriched zOTUs between the normal and IUGR at week 3 and week 4. Specific taxa comparison among groups was analyzed by ANCOM with default setting in qiime2. For the different taxon found with ANCOM, we adopted the Wilcoxon rank test for pairwise tests, adjusted by the Benjamini–Hochberg procedure. The Pearson correlation analysis implemented in Rhea [30] was conducted between relative abundance of genera and the phenotype data after centered log-ratio data transformation. The correlation pairs with adjusted *p*-value < 0.05 were plotted in a scatter plot with linear regression using the default setting of Rhea. The production results were analyzed in SAS (GLM procedure of SAS; SAS Inst. Inc., Cary, NC, USA), testing the difference between IUGR and normal piglets in the model. Means were separated using the PDIFF option and presented as least square means ± SEM and considered significant when *p*  <  0.05 and a tendency when *p*  <  0.10.

## 3. Results

### 3.1. Production Results

The birth weight of normal piglets was 1.51  ±  0.045 kg and for IUGR piglets was 0.77  ±  0.016 kg (*p* < 0.001), and the body weight at day 24 (sampling time) was 6.84  ±  0.313 kg and 4.53  ±  0.174 kg for normal and IUGR piglets, respectively (*p* < 0.001), as presented in Amdi et al. [24]. Normal piglets had an average daily gain (ADG) of 222 ± 19 g/day and IUGR piglets had an ADG of 157 ± 14 g/day (*p* < 0.001).

### 3.2. Gut Microbiota (GM) 

**Figure 1:**
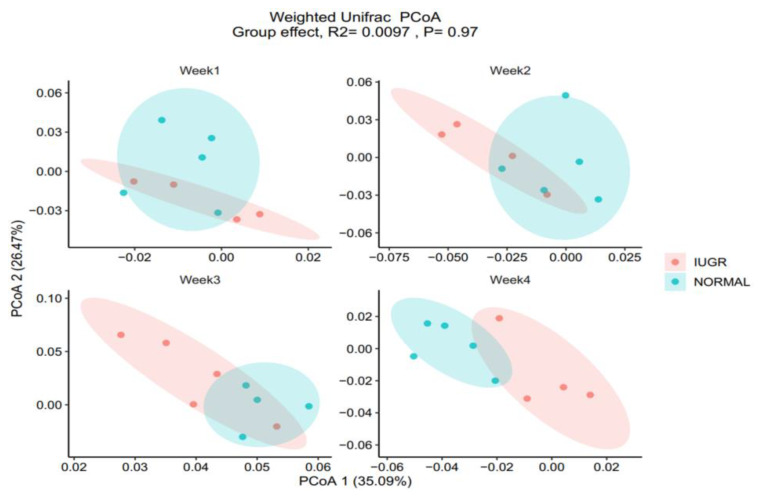
Gut microbiota (GM; as determined by V3 16S rRNA gene amplicon sequencing) of the intrauterine growth-restricted (IUGR) and normal piglet colon content for weeks 1, 2, 3, and 4. Each week, five IUGR and five normal piglets were sacrificed for the study: principal coordinate analysis (PCoA) plots of week 1 weighted Unifrac, *p* = 0.414; week 2 weighted Unifrac, *p* = 0.442; week 3 weighted Unifrac, *p* = 0.268; week 4 weighted Unifrac, *p* = 0.048.

No significant differences between normal and IUGR piglets were detected in alpha diversity measures (Shannon Index of 0.594). Similarly, no significant differences were detected in beta diversity (weighted Unifrac, *R* = 0.0097, *p* = 0.96; unweighted Unifrac, *R* = 0.02, *p* = 0.81) when comparing all IUGR (*n* = 17) and normal piglets (*n* = 19). Although the gut microbiota did not differ between the IUGR and normal piglets, when investigating combined piglet litters at all 4 weeks, we could detect differences between the IUGR and normal piglets for week 3 (IUGR, *n* = 5; normal, *n* = 4) and week 4 (IUGR, *n* = 4; normal, *n* = 5), suggesting a litter/week effect. The comparison of IUGR and normal piglets with regard to beta diversity identified differences for week 3 on unweighted Unifrac metrices, whereas the piglet groups differed both on weighted and unweighted metrices at week 4 (*n* = 5, normal; *n* = 4, IUGR). Figure 1 displays weighted Unifrac PCoA plots of the normal and IUGR GM samples for each week. No separation was found for week 1 and 2 (week 1 weighted Unifrac, *p* = 0.414; unweighted Unifrac, *p* = 0.86; week 2 weighted Unifrac, *p* = 0.442; unweighted Unifrac *p* = 0.22), whereas separation between the groups was found for week 3 weighted Unifrac, *p* = 0.268, and unweighted Unifrac, *p* = 0.041, and week 4 weighted Unifrac, *p* = 0.048, and unweighted Unifrac, *p* = 0.037. Furthermore, we found that the piglets differed on both Unifrac distance metrics at different weeks, indicating that there might have been a sow effect (week) (see Table 1).

**Figure 2:**
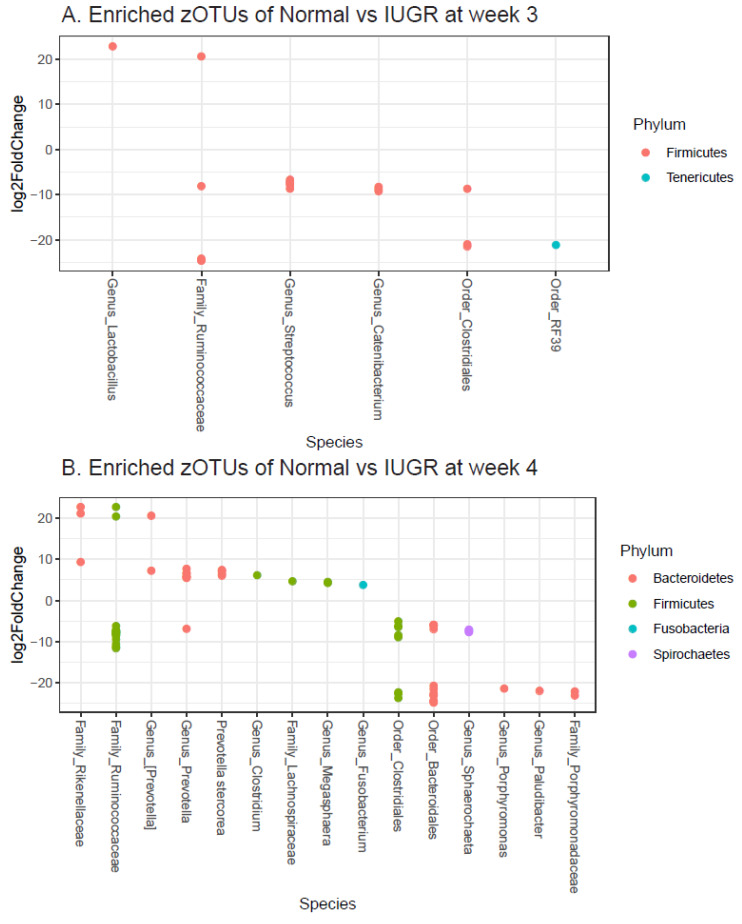
(**A**,**B**) Statistically enriched zero-radius operational taxonomic units (zOTUs) in normal and intrauterine growth-restricted (IUGR) at week 3 (**A**) and week 4 (**B**). Log2Foldchange represents the abundance fold change of normal piglets with respect to IUGR after log2 transformation.

The differences in relative abundance of zOTUs in IUGR and normal piglet samples for week 3 and 4 are displayed in Figure 2A. Figure 2B displays the zOTUs of the IUGR and normal piglets at weeks 3 and 4, for which we identified significant differences between the groups but had no consensus. When looking at the GM of the IUGR and normal piglets at week 4, we found significant differences (adjusted *p* < 0.01) by Deseq2 in the abundance of several zOTUs across the group, with the exception of one of the IUGR samples due to inadequate library size (right side IUGR group). At week 4, the IUGR samples displayed an elevated relative abundance of some zOTUs belonging to the order Bacteroidales, as well as the order Clostridales, and some belonging to the family of Ruminococcaceae, among a few others (*Prevotella*, family Porphyromonadaceae) (Figure 2A,B), whereby the normal piglets displayed other zOTUs at a relatively elevated level, though not across all weeks. We also investigated correlations of GM with bodyweight and did not identify any significant correlation.

### 3.3. Colon Color and GM 

**Figure 3:**
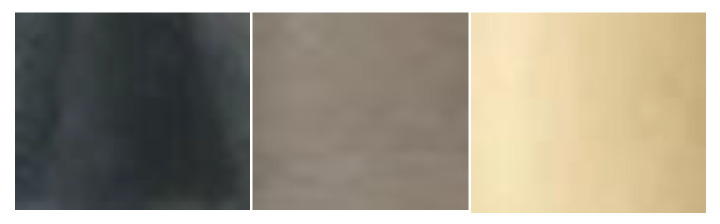
A zoomed view into the colon content: sample 26 (black), sample 35 (yellow, homogenized), and 31 (brown, homogenized).

When dissecting the colons of the piglets and sampling the colon content, we found it to be evident that the piglets fell into three different color categories of colon matter—black, brown, and yellow (Figure 3). Figure 3 depicts the color of the colon content when zoomed into during the preparation of colon content for DNA extraction (see the Supplementary Materials for pictures of sample tubes with samples content). 

**Figure 4:**
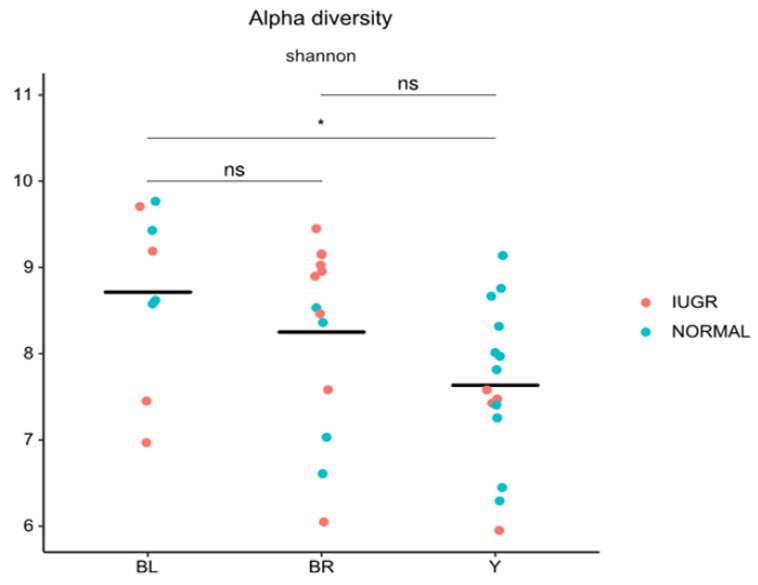
Alpha diversity identified in yellow, brown, and black colon content of normal and intrauterine growth-restricted (IUGR) piglets at weaning.

**Figure 5:**
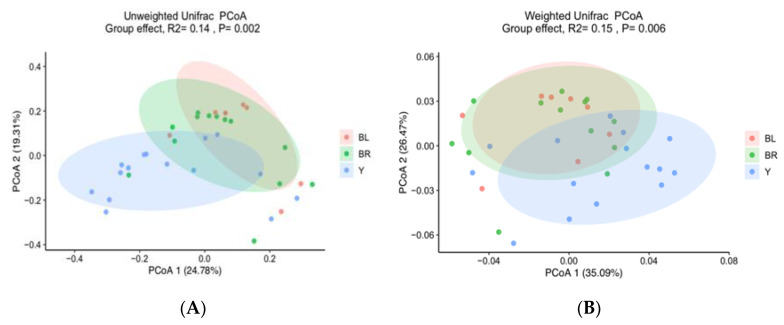
PCoA plot based on weighted (**A**) and unweighted (**B**) Unifrac matrices. Each dot represents the GM community of one sample grouped according to colon content color—yellow, brown, and black.

**Figure 6:**
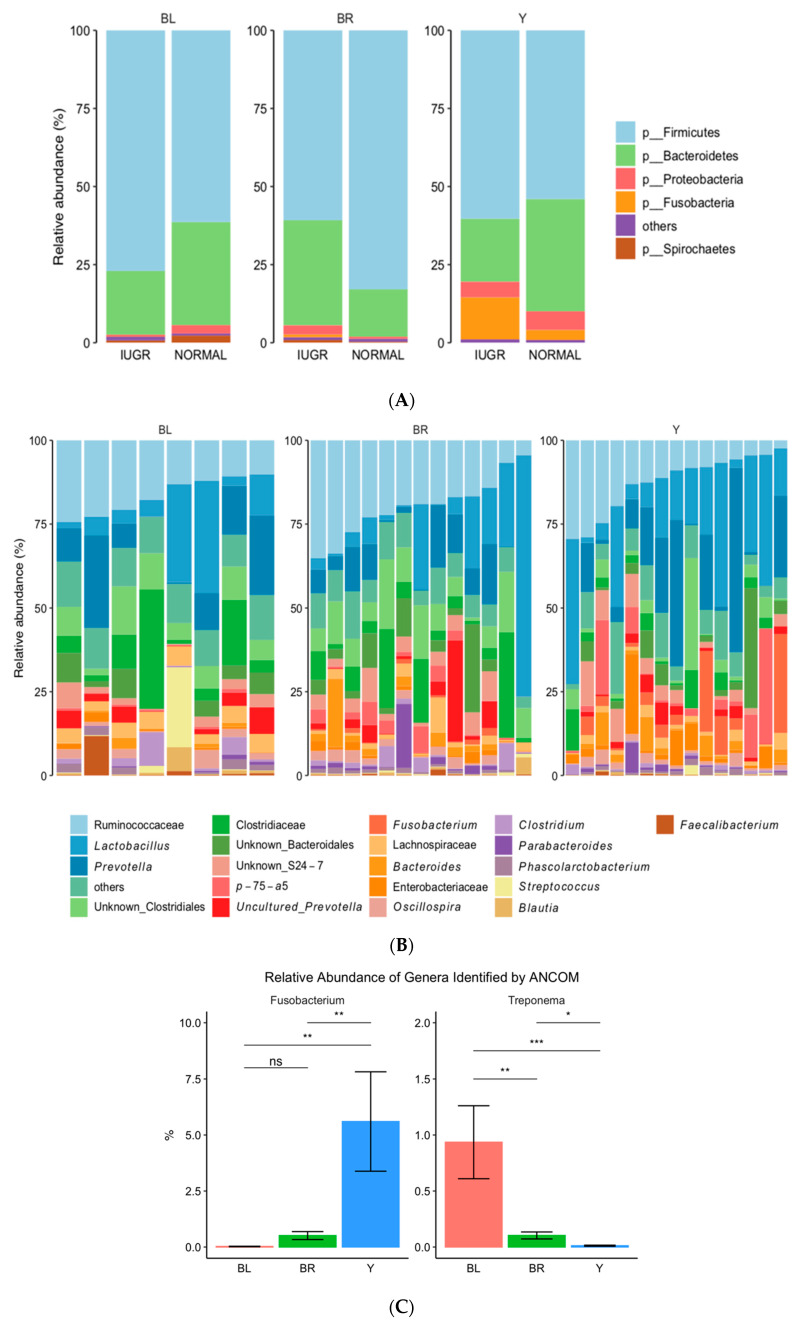
(**A**) Phyla distribution present in yellow (Y), brown (BR), and black (BL) fecal samples (intrauterine growth-restricted (IUGR) and normal); (**B**) distribution of genera across yellow, brown, and black fecal samples; (**C**) overview of the relative abundance of genera present in yellow, brown, and black colon content, as determined by 16S rRNA gene V3-region amplicon sequencing. * indicates a significance at *p* < 0.05, ** indicates a significance at *p* < 0.01 and *** indicates a significance at *p* < 0.001.

The colon microbiome of yellow colon content was found to be significantly less diverse than black content, as determined by Shannon’s diversity index (Figure 4). Similarly, the colon microbiota community composition of yellow samples were significantly different from both black and brown colon content samples (unweighted UniFrac, *R* = 0.14, *p* = 0.001, and weighted Unifrac, *R* = 0.14, *p* = 0.013) (Figure 5A,B). Two genera were identified to significantly contribute to this separation, as indicated in Figure 6A–C. Fusobacterium displayed a significantly higher relative abundance in the yellow colon content, whereas Treponema was significantly more abundant in black colon content.

Solely looking at the grouping of the colon content according to color, we observed via ANCOM significantly more Fusobacteria on the phyla level in the yellow colon content compared to the brown and black colon content (Figure 6A). Figure 6B,C displays the difference in genera composition of the GM belonging to the yellow, brown, and black fecal samples. The relative abundance of two OTUs of the genera Fusobacterium and Treponema were identified by ANCOM as differing significantly in the colon content of different colors (Figure 6C). As for log scale-transformed counts of zOTUs classified with top hits from Ez taxon for Fusobacterirum and Treponema, when all samples were rarefied to 65,000 and log ratio transformed via the Top Hits Ez Taxon Using ezTaxon, we found that the Fusobacterium, which differed significantly, might belong to an unknown species of the Fusobacterium or of *Fusbacterium mortiferum, perfoetens,* or *varium*. For Treponema, most hits were also acquired for an unknown genus of Treponema, besides that a few hits for *Treponema porcinum* and *brennaborense*, as well as the *suis* species.

**Figure 7:**
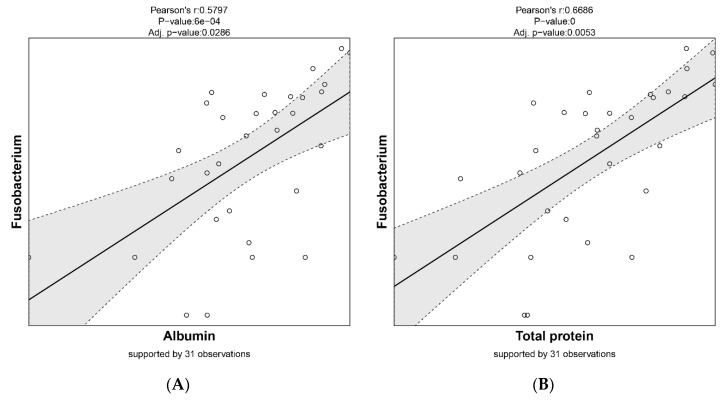

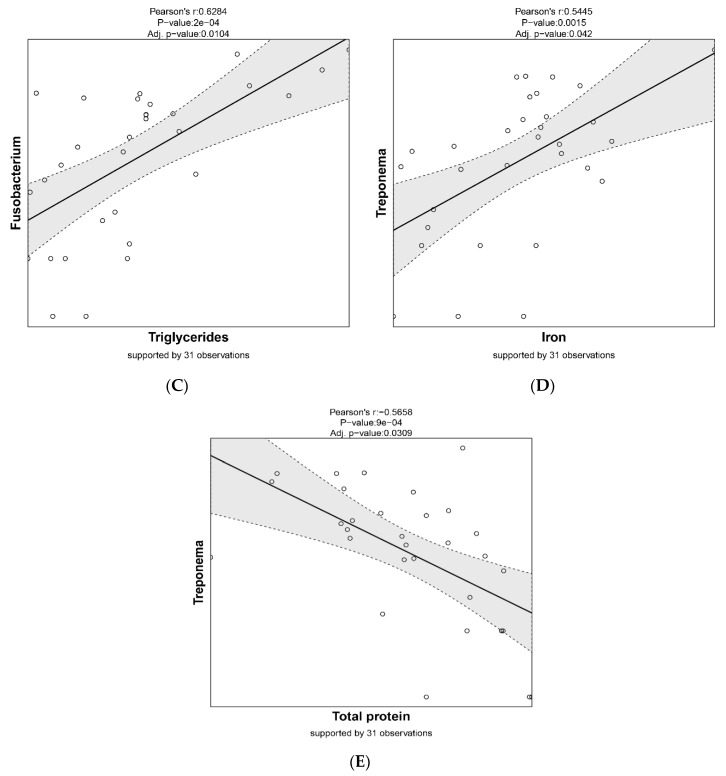
Correlation of significantly different taxa with blood parameters: (**A**) *Fusobacterium* and albumin, (**B**) *Fusobacterium* and total protein, (**C**) *Fusobacterium* and triglycerides, (**D**) *Treponema* and iron, (**E**) *Treponema* and total protein (cut-off for scatter plot was *p*-value < 0.05 and coefficient > 0.5).

The relative abundances of these genera were subsequently correlated to all measured parameters in Amdi et al. [24] (see Figure 7). We identified a significant correlation for a few parameters. *Fusobacterium* positively correlated with total blood protein (TP; *p* = 0.005), albumin (*p* = 0.02), and triglycerides (*p* = 0.01), whereas *Treponema* was most abundant in black colon content, correlating positively with iron (*p* = 0.04) but negatively with total protein (*p* = 0.03) (Figure 7). 

## 4. Discussion

Understanding the composition of the microbial community and its functional capacity during weaning is important for pig production, as bacteria play important roles in pigs’ health and growth performance. Currently, limited information is available regarding the composition and function of the GM of piglets in early life [31]. IUGR is also a large problem in pig production, affecting approximately 15–20% of piglets [5,6,32], and the GM of IUGR piglets is, as of yet, not well understood. In this study, we investigated the gut microbial communities of normal and IUGR piglets. When solely looking at the microbiota identified from the normal piglets, we found community compositions similar to those reported by others. The microbiota of the healthy piglets displayed a proportion of phyla around 50–80% of Firmicutes, 20–48% of Bacteroidetes, 0.1–4% Proteobacteria, and 0–17% Fusobacteria, and thus the composition is comparable to that reported by others around the same age after weaning [33,34].

In this study, we found no significant differences in alpha and beta diversity of the GM sampled from the colon of freshly weaned piglets grouped into the IUGR (*n* = 20) and normal piglets (*n* = 20) and sacrificed over the course of 4 weeks. Recently, Zhang et al. [14], who investigated the microbiota present in the small intestine, reported significant differences in the GM of IUGR and normal piglets (when investigating 48 piglets from 24 litters). Zhang et al. [14] selected piglets on the basis of average litter weight, and IUGR piglets were in their study classified as being within the 10% lower mean birth weight [14]. The piglets in the study by Zhang et al. [14] were not necessarily IUGR. In comparison, the normal piglets in the current study were somewhere in between the IUGR and normal piglets used by Zhang et al. [14]. Danish sows are highly hyperprolific, and the average birth weight is lower than in many other countries [32]. By selecting piglets by the head morphology as done in our study, we selected for piglets that had been subjected to brain sparing and thereby true intrauterine growth restriction, as discussed in [35].

Additionally, our study differed from the study of Zhang et al. [14], as they identified differences in alpha and beta diversity using the 16S rRNA sequencing (V3-V4), while we used the V3-region in this study. They investigated alterations of small intestinal microbiota in IUGR piglets of 7, 21, and 28 days of age, whereas we investigated the changes at day 24. The difference in experimental approach—V3 versus V3 and V4, colon versus small intestine, and piglet age are likely to lead to differences in findings when comparing studies with regard to the findings on a correlation of IUGR with a gut microbial composition [14]. 

The less diverse small intestinal microbiota exposed to different physiological conditions of the host might be more affected by the physiological changes accompanied by IUGR than the more diverse and dense gut microbial community in the large intestine. However, when analyzing the batch per week (week, *n* = 4), we could identify some separation, which might indicate that the experimental design can be improved. For example, differences between batches are most likely also linked to the influence by the sow, i.e., how well she milks, quality of passive immunity, etc., and how much feed next to the sow the piglets consume. This is difficult to quantify, as there can be large variations between pens [36]. No significant correlations of the GM with bodyweight was observed. 

Stool color is a potential indicator of the health status at early age, e.g., reflecting bile flow and similar physiological factors [17]. It is still not well understood as to which extend insights might be gained from investigating stool color also in relation to the microbial composition at early life. Che et al. [37] found differences in stool color of piglets that had been orally inoculated with human fecal suspension compared to piglets that had been orally inoculated with pig fecal suspension where the human flora-associated piglets produced feces that was yellow compared to the pig flora-associated pigs that produced feces that was dark grey [37]. This might be an interesting avenue to be explored for infant and weaner feces. At older age, a complex diet might be masking color to health correlations. In farming, fecal color might be a useful visual indicator for piglet health for farmers and is hence worthwhile exploring. Color of colon content can be a reflection of food and feed, wherein various shades of brown are considered normal. The reason why stool is brown is because of normal production of bile; if there is a problem with bile flow, this may mean a problem in the bile ducts or liver, while some colors are not considered normal, such as black and yellow. Black stool could indicate bleeding in the stomach or the first part of the small intestine, and iron supplements can darken the stool to more of a dark green [38]. Bright red stool usually suggests that blood is coming from the lower part of the digestive system, such as the large intestine, rectum, or anus. Pale white or yellow stool can also signify a problem. Color changes in stool are known to be mostly associated with dietary changes [38,39]. In the current study, it was most likely a mixture of health and dietary changes. Piglets had access to a milk cup system, creep feed, as well as nursing at the sow, all factors that could potentially influence changes in stool color. 

Two genera were identified as significantly contributing to this separation, as indicated by Figure 6. *Fusobacterium* displayed a significantly higher relative abundance in the yellow colon content, whereas *Treponema* was significantly more abundant in the black colon content. We then correlated these genera to all measured parameters and identified significant correlations for a few parameters. *Fusobacterium* positively correlated with total blood protein, albumin, and triglycerides, whereas *Treponema*, most abundant in black colon content, correlated positively with iron (Figure 7).

*Treponema* was most abundant in the black feces and is a genera that is associated with infections in IUGR piglets [40], although it was not elevated in IUGR piglets in this study. To further support these results, no differences were found in the blood biochemistry between IUGR and normal piglets, for example, the albumin levels (34.9 g/L vs. 34.5 g/L; *p* = 0.796) and total protein levels (49.95 g/L vs. 47.65 g/L; *p* = 0.207) were similar in normal and IUGR pigs, respectively [24]. 

It has been reported that the genera Fusobacterium is closely correlated with diseases in animals [40] and it is likely that pathobiont species are commonly present in the infant or inchoate piglet gastrointestinal tract. Because inchoate piglets are highly susceptible to various diseases, whether the proliferation of these potential pathogens has negative impact on piglet health warrants further research [40]. It needs to be further investigated as to whether yellow fecal color is consistently correlated with a relatively high abundance of *Fusobacterium*, and if this association is somewhat relevant for piglet health. In the future, it would be also interesting to evaluate the correlation of fecal color with the eukaryotic fraction of the microbiome including fungi. The increased level of blood protein that correlated with *Fusobacterium* might be indicative for an inflammatory response, and hence disease. As there is currently a large focus on reducing both antibiotic and zinc oxide usage in pig production, further refinement of the relationship between stool color and intestinal health may be a novel method for rapid, on-farm determination of piglets that require treatment. This may play a role in more efficient and sustainable use of antimicrobials to promote health and performance in young pigs. 

## 5. Conclusions

In conclusion, no significant differences were detected in colon microbiota composition between IUGR and normal piglets, but we observed that colon content had one of three different colors, namely, brown, black, and yellow. The color of the colon content varied with microbial community composition, with significant differences in the relative abundance of taxa belonging to the genera Fusobacteria and *Treponema*. Fusobacteria were most abundant in yellow fecal samples and positively correlated with total blood protein, albumin, and triglycerides. Contrarily, *Treponema* was most abundant in black fecal samples and correlated positively with blood iron content. This study indicates that colon/fecal content color can possibly be used as indicator for specific GM and metabolite signatures. Further research is required to confirm if assessment of stool color may play a role in diagnosis and control of intestinal disease and dysfunction. 

## Figures and Tables

**Table 1:** Numbers of intrauterine growth-restricted (IUGR) and normal piglet colon content of different colors.

Piglet	Yellow	Black	Brown
IUGR	5	4	11
Normal	11	4	5
Total number	16	8	16

## Supplementary Materials

The following are available online at https://www.mdpi.com/2076-2615/10/6/1073/s1; Table S1: Unifrac distance metrics at different weeks and Figure S1: Examples of colon content samples in falcon tubes.
